# Self-Assisted Finger Stiffness Splint (SFSS)

**DOI:** 10.7759/cureus.51921

**Published:** 2024-01-09

**Authors:** Ahmad Almigdad, Naseem Obeidat, Muna Melhem, Saba’a Abu-Ashour

**Affiliations:** 1 Department of Orthopedics, Royal Medical Services, Amman, JOR; 2 Department of Occupational Therapy, Royal Medical Services, Amman, JOR

**Keywords:** tenolysis, occupational therapy, flexion contracture, splint, finger stiffness

## Abstract

Finger stiffness may arise from injuries, surgeries, or hand-related medical conditions, impacting hand function and overall well-being in daily life. Rehabilitation and hand therapy play a crucial role in restoring optimal range of motion, strength, and functionality. This article introduces the Self-Assisted Finger Stiffness Splint (SFSS), a dynamic splint designed for active finger movement applicable in post-trauma or postoperative rehabilitation. SFSS empowers patients to perform self-administered stretching exercises, expediting recovery and improving compliance. Its versatility extends to postoperative rehabilitation, covering cases like tenolysis of extensor tendons or rehabilitation after fracture healing. While particularly effective for proximal interphalangeal joint (PIPJ) and metacarpophalangeal joint (MCPJ) stiffness, SFSS remains valuable for managing isolated finger stiffness and proves beneficial in addressing multiple-digit stiffness.

## Introduction

Finger stiffness is a condition characterized by a reduced range of motion in the fingers, making it challenging to fully flex or extend them. This condition can impact one or multiple fingers and may be associated with various underlying causes, including congenital, traumatic, inflammatory, and systemic diseases such as scleroderma. Stiffness may originate from any anatomical structure within the hand and fingers, encompassing the skin, tendons, bones, and joints. The severity of symptoms related to finger stiffness can vary from mild discomfort to intense pain, contingent on the specific underlying cause [1, 2].

The clinical examination should check the stability and flexibility of the skin, fascia, and neurovascular structures. It is necessary to evaluate both passive and active movements in each joint. If passive movement exceeds active movement, it may indicate issues with muscles or tendons. Conversely, if active and passive movements are equal, the problem could be within the joint, related to bones, or involve capsuloligamentous fibrosis. If the fingers cannot fully extend while bending the wrist, it might be due to tight muscles or adhesions. The Bunnell test helps identify tightness in intrinsic muscles, with a positive result showing reduced proximal interphalangeal joint (PIPJ) flexion during metacarpophalangeal joint (MCPJ) extension compared to MCPJ flexion. Hand radiographs are essential to rule out bone-related problems [3, 4].

Preventing stiffness through proper immobilization and early rehabilitation is a fundamental principle, but addressing established stiffness poses challenges. Potential treatment approaches encompass physical therapy, anti-inflammatory medications, splinting, and, in certain instances, surgical intervention. For intricate cases involving contracture in multiple fingers, it is advisable to refer patients to specialized centers where repeated local anesthesia can facilitate therapy and splint adjustments. Hand therapy progresses through a systematic approach, including splint use, active motion exercises, electrical stimulation therapy, and skin gliding work. Occupational therapy plays a crucial role in reintegrating the movement of stiff fingers into overall hand motions, with a collaboratively established schedule outlining exercises, attained range of motion, pain triggers, and recovery of sensibility for fingers with nerve defects. Regular reassessment and adjustments to the treatment plan are vital components [5].

Operative intervention is recommended when nonoperative treatment fails. Surgery typically addresses osseous and articular pathology; such as fracture malunion or exostoses. Joint replacement is more effective for alleviating pain than addressing stiffness. Successful surgery and early rehabilitation necessitate a stable soft-tissue covering; scar contractures may necessitate excision and tissue reconstruction. Contracted skin or fascia limiting motion is addressed with release, fasciotomy, or resurfacing. Disrupted extensor mechanisms or flexor tendon adhesions may require tenolysis, rebalancing, or reconstruction, potentially involving capsuloligamentous releases [6].

In this article, we introduce a splint designed for active movement of fingers, offering potential applications post-trauma or as part of postoperative treatment. Termed the Self-assisted Finger Stiffness Splint (SFSS), this splint provides self-exercises for managing finger stiffness.

## Technical report

Orthopedic splints are crucial for treating various conditions of the hands and fingers. The purposes of a splint include immobilization, mobilization, restraining, or supporting various body parts. The materials used in manufacturing orthopedic splints should possess specific characteristics to ensure their effectiveness. Typically, materials like thermoplastic, fiberglass, or plaster are utilized due to their moldability and rigidity when set. This enables clinicians to create custom-fitted splints tailored to individual patient needs. The choice of material is key, considering factors such as flexibility, durability, and radiolucency, allowing for proper assessment without the need for removal. Carefully selecting splint materials in orthopedic care is fundamental to achieving optimal therapeutic outcomes. We utilize thermoplastic material in the introduced splint in this paper, SFSS.

Thermoplastic splints

Thermoplastic splints, commonly formulated from materials like polyethylene, polypropylene, or a blend of these polymers, exhibit unique properties ideal for splinting applications. Their thermoplastic nature allows them to become pliable and moldable when heated, enabling customization for each patient. Notably, their exceptional moldability empowers clinicians to shape the splint directly on the patient for a precise fit. Once cooled, these splints offer remarkable rigidity, providing sturdy support and effective immobilization. Their lightweight nature enhances patient comfort, and the often radiolucent material allows healthcare professionals to assess the affected area without removal. Moreover, thermoplastic splints are durable and resistant to wear, ensuring prolonged and effective use in orthopedic and rehabilitation scenarios [7, 8].

The preparation of thermoplastic splints involves several key steps to ensure proper fit, functionality, and patient comfort. Initially, thermoplastic sheets are heated using a dry heat source, such as a heat gun or warm water bath, until they become pliable. In this softened state, clinicians mold the material directly onto the patient's body part, using their hands or molds to achieve the desired shape and fit. Subsequently, the molded splint is allowed to cool and harden, securing its intended shape, a process that can be expedited using cold water or air. After cooling, excess material is trimmed, and edges are smoothed to enhance comfort and aesthetics. Additional components, such as Velcro straps or padding, may be added to improve fixation and overall comfort during use. The final step involves a thorough evaluation of the fit, comfort, and immobilization provided by the thermoplastic splint, ensuring its effectiveness in clinical orthopedic and rehabilitation applications [8, 9].

Self-Assisted Finger Stiffness Splint (SFSS)

**Figure 1:**
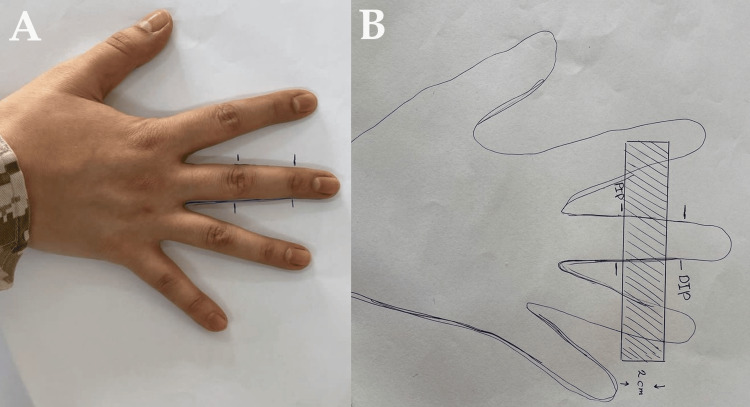
Drawing the model on the thermoplastic sheets A: Using the patient hand to draw the model to customize the splint. B: Preparing the model on a thermoplastic sheet. PIP: proximal interphalangeal joint; DIP: distal interphalangeal joint

This splint allows the patient to actively mobilize a stiff finger using adjacent fingers of the same hand. Therefore, it is more effective in isolated digital stiffness. However, it may be useful in multiple finger stiffness. This model is useful for all fingers except the thumb. This splint is applied on the middle phalanx to provide a longer lever arm and allow mobilization of both PIPJ and MCPJ (Figure 1). However, it can be applied over the proximal phalanx, but this allows movement at MCPJ only and is less efficient secondary to a shorter lever arm of the movement.

**Figure 2:**
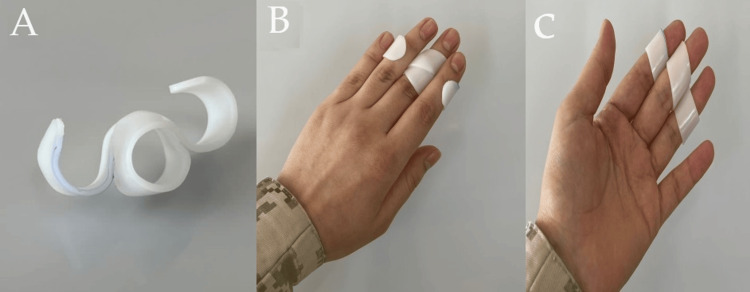
Middle finger Self-Assisted Finger Stiffness Splint (SFSS) A: The proposed model for the middle finger, the ring around the index and ring finger, can be completed to a full ring or kept open. B, C: Middle SFSS dorsal and volar view. Note the different levels of splint to fit the middle phalanx at the index and ring finger. Flexion force from the index and ring fingers is used to bend the middle finger at the proximal interphalangeal joint (PIPJ) and proximal interphalangeal joint (PIPJ), including the index and ring finger, to control rotation.

**Figure 3:**
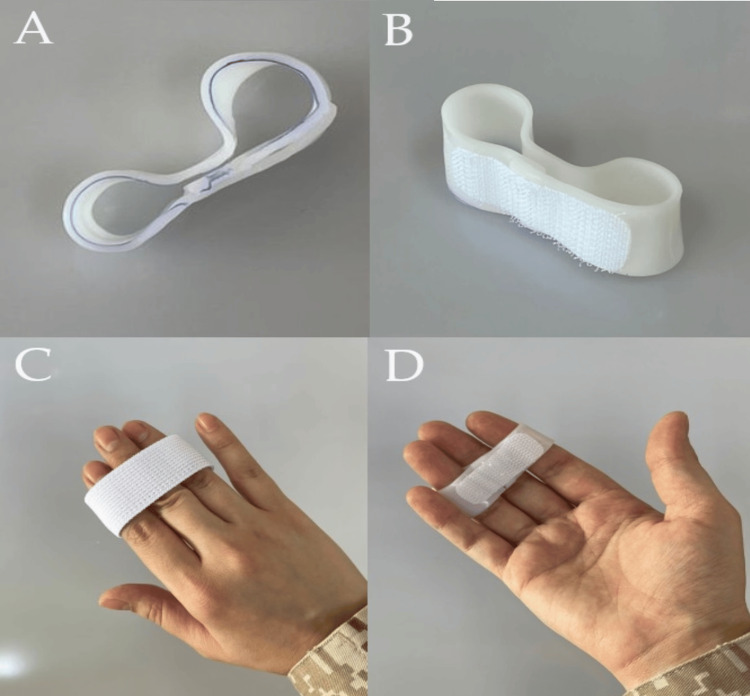
Index and ring finger Self-Assisted Finger Stiffness Splint (SFSS) A, B: The proposed index and middle finger splint, different views; similarly, the ring around the middle finger can be completed to complete the ring. Including three fingers to control rotation and make the splint more efficient. C, D: Dorsal and volar view of index and ring SFSS, with the adjustment for splint to fit a patient finger over the middle phalanx.

The splint should be applied on three adjacent fingers to control rotation and to provide more controlled movement. Models involving two fingers were tried but were less efficient, less comfortable, and did not prevent rotation. Therefore, we use a model including three adjacent fingers. Due to the different lengths of fingers, the splint should be adjusted to fit the middle phalanx of the three adjacent fingers. The splints for the index, middle, and ring fingers are nearly the same (Figures 2, 3).

**Figure 4:**
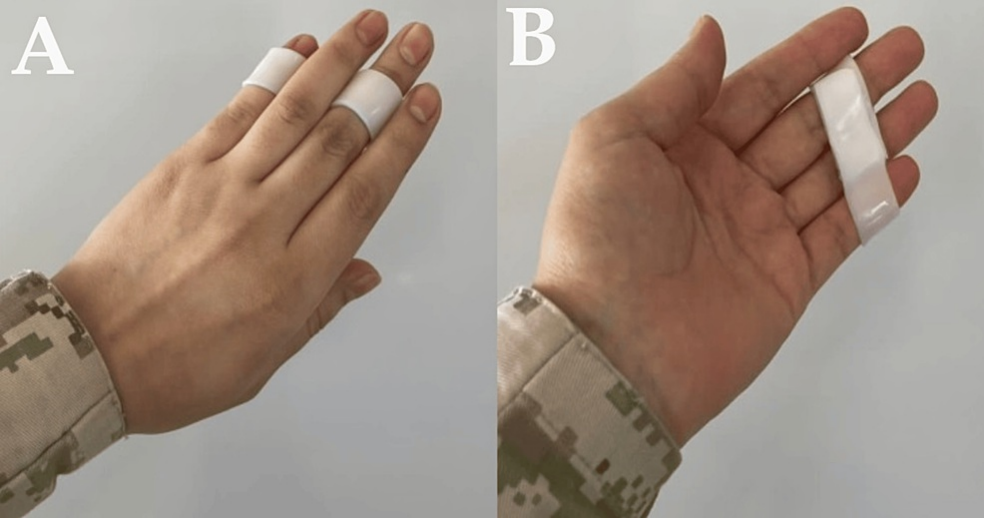
Little finger splint A: Dorsal view. B: Volar view. Because of the unleveled middle phalanx of the little finger with ring and middle, the splint was adjusted to fit the middle phalanx of the middle, ring, and little fingers.

However, the little finger needs a modified splint to fit it on the middle phalanx because of its shortness, Figure 4.

The splint can be used by the patient frequently during the day. It allows stretching of the stiff fingers and allows easy and self-delivered stretching exercises of the stiff fingers, which achieve faster recovery. Such a splint is associated with more convenience and compliance than self-exercises alone.

SFSS is useful in rehabilitating a stiff finger after surgery, such as tenolysis of extensor tendons, and it can be used immediately if the wound is not extending over PIPJ or waiting until the wound is healed. In case of fracture, it can be applied after fracture healing. While SFSS is useful for PIPJ and MCPJ, it is not useful in distal interphalangeal joint (DIPJ) stiffness. Additionally, a splint is more effective in isolated finger stiffness but is still useful in multiple-digit stiffness.

## Discussion

Finger stiffness often arises as a common concern following various injuries, surgeries, or hand-related medical conditions. The resultant restriction in finger mobility significantly impacts daily activities, leading to functional limitations and reduced quality of life. Addressing finger stiffness through rehabilitation is essential, aiming to restore optimal range of motion, strength, and overall functionality. This comprehensive process involves a multidisciplinary approach, combining therapeutic exercises with patient education to promote recovery [1,2,5].

The treating physician routinely refers patients experiencing finger stiffness to the occupational therapy team, where they undergo comprehensive evaluations. A customized rehabilitation program is meticulously devised, incorporating therapeutic exercises, splinting, and educational components. Emphasis is placed on instructing patients on the proper use of tools, such as splints, a critical aspect of the rehabilitation process.

Patients are strongly encouraged to continue their rehabilitation independently at home through prescribed exercises. However, compliance with home exercises can be challenging, often due to perceived difficulty or issues with multiple instructions. Addressing these compliance barriers is integral to the success of the rehabilitation program. A primary clinical strategy involves adapting home exercises to enhance patient convenience and comfort, potentially involving simplifying instructions and additional support to boost adherence.

The introduction of SFSS proves instrumental in facilitating stretching exercises and fostering patient compliance. SFSS, crafted from readily available and cost-effective thermoplastic materials within the occupational therapy department, serves as a customized and easily modifiable splint. Its utility in fitting patient fingers makes it a valuable tool for effectively rehabilitating patients with stiffness.

We found a close design to SFSS but with a different indication and application called Relative Motion Extension Orthosis (RMEO).

Relative Motion Extension Orthosis (RMEO)

**Figure 5:**
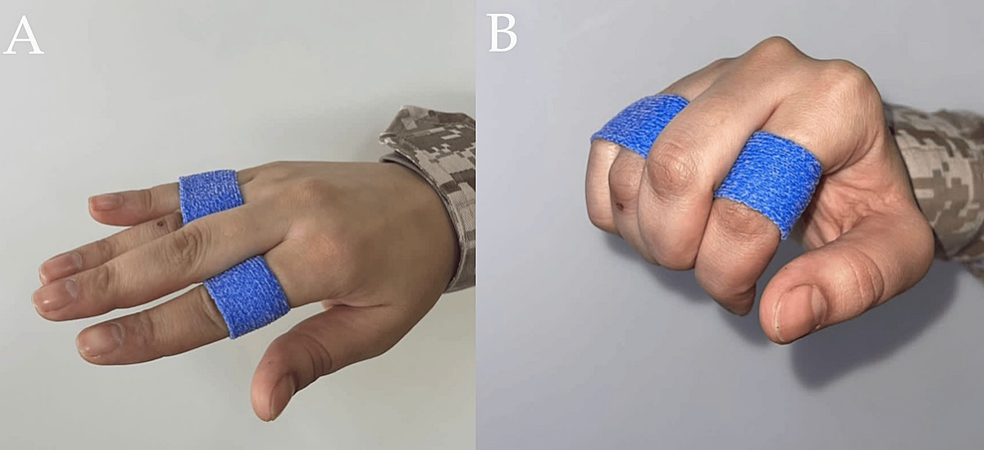
Yoke splint for the middle finger This splint permits flexion lag compared to other fingers during flexion of fingers at the metacarpophalangeal joint (MCPJ) to protect extensor tendon repair during rehabilitation. A: With finger extension. B: With finger flexion. Similarly, adjustment of the splint to fit the proximal phalanx is needed.

A Relative Motion Extension Orthosis (RMEO), also known as a yoke splint, is typically crafted from thermoplastic material. It positions the affected finger in a relative extension of 15 to 20 degrees at the metacarpophalangeal joint, in contrast to adjacent fingers that share the same common extensor muscle belly. By limiting the finger's flexion compared to neighboring fingers, it reduces the force exerted on the mended tendon in comparison to adjacent tendons, resulting in approximately 5 mm decreased tendon excursion (Figure 5). This design ensures secure, nearly full digital motion, preventing stress that could lead to the rupture of the repaired tendon [10]. Although the design is similar to SFSS, the indication and application differ. A yoke splint is applied over the proximal phalanx and aims to protect extensor tendon repair by keeping the operated finger in a more extended position.

We believe SFSS is beneficial for rehabilitating stiff fingers, enabling patients to perform active-assisted exercises using adjacent fingers, making it easy to use. While particularly efficient for isolated finger stiffness, its value extends to cases of multiple finger stiffness due to the cumulative power strength effect from actively moving the splinted three fingers simultaneously.

## Conclusions

Finger stiffness, originating from various causes, significantly impacts daily life. Effective rehabilitation is crucial, and the SFSS provides a practical solution. SFSS allows patients to actively manage finger stiffness with convenient self-administered stretching exercises, promoting faster recovery and better compliance. Its versatility extends to postoperative rehabilitation, addressing conditions like tenolysis of extensor tendons or rehabilitation after fracture healing. While especially effective for PIPJ and MCPJ stiffness, SFSS is more useful for isolated finger stiffness but is still a valuable tool in managing multiple-digit stiffness.
